# Archaeal and Bacterial Metagenome-Assembled Genome Sequences Derived from Pig Feces

**DOI:** 10.1128/mra.01142-21

**Published:** 2022-01-20

**Authors:** Matthew Crossfield, Rachel Gilroy, Anuradha Ravi, David Baker, Roberto M. La Ragione, Mark J. Pallen

**Affiliations:** a University of Bath, Claverton Down, Bath, United Kingdom; b Quadram Institute Bioscience, Norwich Research Park, Norwich, United Kingdom; c School of Veterinary Medicine, University of Surrey, Guildford, Surrey, United Kingdom; d School of Biosciences and Medicine, University of Surrey, Guildford, Surrey, United Kingdom; e University of East Anglia, Norwich Research Park, Norwich, United Kingdom; Portland State University

## Abstract

We report the recovery of metagenome-assembled genomes (MAGs) from fecal samples collected in 2018 from five healthy adult female pigs in southeast England. The resulting nonredundant catalog of 192 MAGs encompasses 102 metagenomic species, 41 of them novel, spanning 10 bacterial and 2 archaeal phyla.

## ANNOUNCEMENT

The domestic pig is one of the most common livestock animals in the world. However, taxonomic and genomic diversity within the pig gut microbiome remains largely unexplored. We therefore applied metagenomic sequencing and analysis to five fecal samples from pigs raised on a commercial livestock farm in Surrey, UK, without the use of antibiotic supplements.

Ethical approval was obtained from the University of Surrey’s Animal Welfare and Ethical Review Body under agreement NERA-2018-011. DNA was extracted from five freshly voided fecal samples using the PowerSoil DNA isolation kit (MoBio Laboratories, Inc., CA, USA) before library construction using the Nextera XT library preparation kit according to the manufacturer’s recommendations. The final pool quality was assessed using the Agilent 2200 TapeStation system and the concentration quantified using Qubit v4. A total of 243,575,779 paired-end reads (2 × 150 bp) were generated on the Illumina NextSeq platform. All subsequent bioinformatics tools were run with default parameters unless otherwise specified. The read quality was assessed using FastQC v0.11.8, before mapping to the host genome (GenBank accession no. GCF_000003025.6) for depletion of the host reads using Bowtie2 v2.3.4 and SAMtools v1.9.0 (1–5) (https://doi.org/10.6084/m9.figshare.16896877.v3).

Sample-specific assemblies were generated using MegaHIT v1.0.6 (6), and the host-depleted reads were mapped back to the assemblies using Bowtie2 and SAMtools to determine the coverage. Contigs were binned using MetaBAT 2 v2.12.1 (7) (contig length, ≥2.5 kb), CONCOCT v1.1.0 (8), and MaxBin 2 v2.2.7 (9) (contig length, ≥1 kb). DAS Tool v1.1.2 was used to integrate the bin predictions and create five sets of optimized, nonredundant metagenome-assembled genomes (MAGs) (10). CheckM v1.0.13 (11) was used to obtain estimates of the completion and contamination, and bins with ≥70% completion, ≤10% contamination, and/or a quality score (completeness minus 5× contamination) of >50 were followed up as “medium- or high-quality MAGs.” MAGs were dereplicated using dRep v2.6.2 (12) at 95% and 99% average nucleotide identity (ANI) (for species and strains, respectively). Taxonomic assignments were made using the Genome Taxonomy Database Toolkit v1.5 (GTDB-tk) with the release 202 database (13). Proteomes were predicted using Prodigal v2.6.1 (14) before comparison against 400 universal marker proteins using PhyloPhlAn v3.0.58 (15) in accordance with DIAMOND v0.9.34 (16) and using the supermatrix configuration at a high diversity scale. Multiple sequence alignment and subsequent refinement were performed using MAFFT v7.271 and trimAl v1.4 (17, 18), before tree construction and refinement using FastTree v2.1.10 (19) and RAxML v8.2.12 (20), respectively. All trees were visualized and annotated using iTol v5.7 (21).

**FIG 1:**
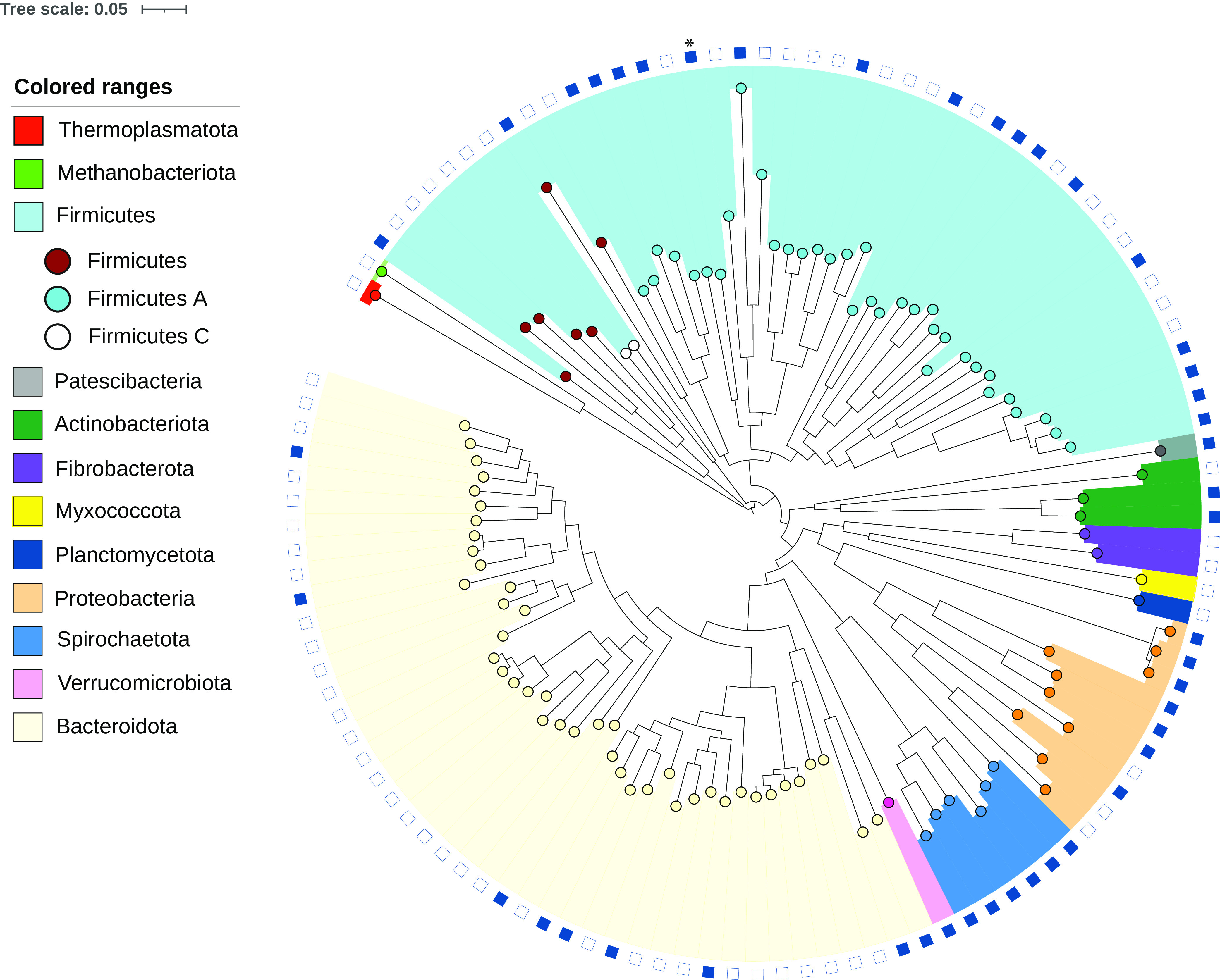
Phylogeny of 114 metagenome-assembled genomes from five pig fecal samples. Computed using PhyloPhlAn with the supermatrix configuration on 400 universal merger genes at high diversity scale; visualized and annotated using iTOLv5.7, with the scale bar depicting internal branch lengths. The phyla assigned by GTDB are shown by color. Branch point colors subdivide the traditional phylum *Firmicutes* into the three phyla assigned by GTDB. Blue filled rectangles represent known species within GTDB, with unfilled shapes representing novel species. The asterisk denotes the placement of the representative of a new family within the order *Christensenellales*.

This workflow resulted in a nonredundant catalogue of 192 MAGs, representing 102 metagenomic species spanning 12 phyla. Forty-one of the species are considered novel, showing no classification within currently available databases, including a representative of a new family within the order *Christensenellales* (Fig. 1; Table 1).

**TABLE 1:** Metagenome-assembled genomes from five pig fecal samples^*a*^

MAG_ID^*b*^	Species/strain	Taxonomic assignment^*c*^	Coverage (×)	FastANI (%)	Genome size (bp)	No. of contigs	*N*_50_ (bp)	GC content (%)	Completeness (%)	Contamination (%)	BioSample accession no.	GenBank accession no. or Figshare link
108^*d*^	1_1	Megasphaera elsdenii	14.6	98.92	2,081,739	458	7,910	54.14	91.38	2.59	SAMN16580179	https://doi.org/10.6084/m9.figshare.16896877.v3
51	1_1	Megasphaera elsdenii	NA	98.92	1,893,361	591	4,166	54.59	93.03	1.88	SAMN16580180	https://doi.org/10.6084/m9.figshare.16896877.v3
12	1_1	Megasphaera elsdenii	NA	98.92	1,898,913	543	4,510	54.53	98.12	1.72	SAMN16580181	https://doi.org/10.6084/m9.figshare.16896877.v3
54^*d*^	10_1	Novel species 10 within the genus W2P13-069	4.4	NA	1,281,580	645	2,152	65.25	79.67	3.61	SAMN16580179	GCA_018383355.1
157_3	10_1	Novel species 10 within the genus W2P13-070	NA	NA	1,364,454	735	1,906	64.84	87.15	6.37	SAMN16580180	https://doi.org/10.6084/m9.figshare.16896877.v3
119^*d*^	10_2	Novel species 10 within the genus W2P13-071	NA	NA	1,502,460	617	2,855	65.19	80.17	4.31	SAMN16580181	https://doi.org/10.6084/m9.figshare.16896877.v3
164^*d*^	100_0	UBA4248 sp004554395	7.3	98.92	3,068,288	706	5,709	52.11	91.38	0	SAMN16580178	GCA_018384915.1
192_2^*d*^	101_0	Novel species 101 within the genus RZZT01	7.1	NA	627,079	166	5,146	42.04	80.56	0	SAMN16580181	GCA_018384895.1
222^*d*^	102_0	RUG369 sp004556055	6.3	98.87	2,737,201	738	4,630	51.12	84.33	1.02	SAMN16580181	GCA_018384795.1
142_2^*d*^	11_1	F23-B02 sp004556755	8.5	99.02	1,554,224	231	9,615	54.92	97.65	6.35	SAMN16580178	https://doi.org/10.6084/m9.figshare.16896877.v3
161	11_1	F23-B02 sp004556755	6.2	99.02	1,455,046	274	6,763	55.2	94.89	0	SAMN16580179	GCA_018385075.1
MB2.57^*d*^	12_0	SFMI01 sp004556155	8.3	99.25	2,129,070	271	9,714	59.08	85.34	2.59	SAMN16580178	GCA_018384115.1
MB2.59^*d*^	13_0	PeH17 sp004556165	121.7	98.31	1,330,707	69	30,851	50.07	86.21	0	SAMN16580179	GCA_018384005.1
MBin.060^*d*^	14_0	PeH17 sp000435055	14.2	98.89	1,884,822	135	24,258	49.6	96.55	2.59	SAMN16580178	GCA_018384705.1
159_2^*d*^	15_0	*Phascolarctobacterium*_A *succinatutens*	24.8	99.42	1,935,117	339	8,719	48.28	89.34	5.17	SAMN16580180	https://doi.org/10.6084/m9.figshare.16896877.v3
MBin.031^*d*^	16_0	Ruminococcus flavefaciens_G	30.8	97.45	2,560,973	95	43,414	51.35	100	0	SAMN16580178	https://doi.org/10.6084/m9.figshare.16896877.v3
123^*d*^	17_0	CAG-180 sp004556705	8.4	98.07	1,673,664	349	6,771	43.71	90.34	6.19	SAMN16580179	GCA_018384955.1
64^*d*^	18_0	Novel species 18 within the genus DTU089	8.1	NA	1,810,698	519	4,294	38.78	98.28	1.88	SAMN16580181	GCA_018384655.1
MB2.42^*d*^	19_0	Novel species 19 within the genus HGM11525	18.0	NA	1,976,368	150	19,416	39.82	93.1	3.45	SAMN16580178	GCA_018384175.1
MBin.003_2	2_1	Novel species 2 within the genus *Comamonas*	NA	NA	2,733,925	206	20,654	60.62	97.93	0	SAMN16580180	https://doi.org/10.6084/m9.figshare.16896877.v3
MB2.19^*d*^	2_1	Novel species 2 within the genus *Comamonas*	28.8	NA	2,672,762	99	45,383	60.77	98.28	0	SAMN16580182	GCA_018384445.1
192^*d*^	20_1	CAG-115 sp004555635	7.0	98.06	2,141,678	498	5,642	52.02	93.5	5.04	SAMN16580178	GCA_018384805.1
87	20_1	CAG-115 sp004555635	NA	98.06	1,969,602	530	4,700	52.29	88.48	1.88	SAMN16580179	https://doi.org/10.6084/m9.figshare.16896877.v3
126^*d*^	21_1	Novel species 21 within the genus *Ruminiclostridium*_E	11.0	NA	1,651,903	724	2,519	44.73	92.79	1.88	SAMN16580182	GCA_018385035.1
46^*d*^	21_2	Novel species 21 within the genus *Ruminiclostridium*_E	NA	NA	1,679,956	712	2,527	45.05	91.22	4.08	SAMN16580181	https://doi.org/10.6084/m9.figshare.16896877.v3
164_2^*d*^	21_3	Novel species 21 within the genus *Ruminiclostridium*_E	NA	NA	1,717,232	858	2,149	44.83	88.32	5.49	SAMN16580180	https://doi.org/10.6084/m9.figshare.16896877.v3
4_3^*d*^	21_4	Novel species 21 within the genus *Ruminiclostridium*_E	NA	NA	1,839,562	671	3,102	44.9	91.22	3.76	SAMN16580178	https://doi.org/10.6084/m9.figshare.16896877.v3
MB2.83^*d*^	22_1	Novel species 22 within the order *Christensenellales*	11.5	NA	1,636,615	80	32,741	57.98	89.66	0	SAMN16580178	GCA_018385275.1
MB2.66_2	22_1	Novel species 22 within the order *Christensenellales*	NA	NA	1,384,958	239	6,702	58.11	75.86	1.72	SAMN16580179	https://doi.org/10.6084/m9.figshare.16896877.v3
MB2.1_2	23_1	“*Angelakisella*” sp004554485	NA	98.33	1,156,090	185	7,110	59.74	85.58	0.16	SAMN16580179	https://doi.org/10.6084/m9.figshare.16896877.v3
MB2.042^*d*^	23_1	*Angelakisella* sp004554485	13.1	98.33	1,192,484	181	7,616	59.78	78.06	1.72	SAMN16580181	GCA_018384415.1
MB2.34_2	23_1	*Angelakisella* sp004554485	NA	98.33	1,112,822	203	6,282	59.61	77.59	0.16	SAMN16580182	https://doi.org/10.6084/m9.figshare.16896877.v3
MB2.127^*d*^	24_1	CAG-272 sp000433515	20.8	98.56	2,059,360	75	41,032	53.15	94.83	0	SAMN16580179	GCA_018383935.1
54_2	24_1	CAG-272 sp000433515	NA	98.56	2,148,591	372	8,222	53.33	96.24	9.09	SAMN16580180	https://doi.org/10.6084/m9.figshare.16896877.v3
MB2.120^*d*^	25_1	Novel species 25 within the genus CAG-841	15.0	NA	1,476,605	90	24,557	49.07	95.72	0	SAMN16580179	GCA_018383975.1
MB2.51	25_1	Novel species 25 within the genus CAG-841	NA	NA	1,345,663	156	10,952	49.1	92.24	0	SAMN16580180	https://doi.org/10.6084/m9.figshare.16896877.v3
MB2.013^*d*^	26_0	Novel species 26 within the genus UBA1712	9.4	NA	1,429,645	186	9,637	44.02	79.15	1.72	SAMN16580181	GCA_018384635.1
MB2.132^*d*^	27_0	UBA2868 sp004552595	10.0	98.84	1,459,382	226	8,072	38.12	87.93	1.72	SAMN16580178	GCA_018384285.1
MBin.045	28_1	Novel species 28 within the genus *Acetatifactor*	NA	NA	2,381,748	366	11,666	50.42	85.52	3.45	SAMN16580178	https://doi.org/10.6084/m9.figshare.16896877.v3
MB2.86^*d*^	28_1	Novel species 28 within the genus *Acetatifactor*	38.3	NA	2,467,536	177	22,051	50.22	94.83	4.48	SAMN16580179	GCA_018385425.1
MB2.96_2	28_1	Novel species 28 within the genus *Acetatifactor*	NA	NA	1,898,589	369	5,714	50.24	75.41	1.72	SAMN16580180	https://doi.org/10.6084/m9.figshare.16896877.v3
MB2.1	28_1	Novel species 28 within the genus *Acetatifactor*	NA	NA	2,322,269	252	12,308	50.45	83.86	4.31	SAMN16580182	https://doi.org/10.6084/m9.figshare.16896877.v3
MBin.029^*d*^	29_0	Novel species 29 within the genus CAG-95	27.9	NA	3,111,859	125	51,022	43.83	95.45	3.45	SAMN16580178	GCA_018384375.1
113	3_1	UBA1723 sp002371265	NA	96.37	3,291,296	456	9,374	41.24	94.83	1.72	SAMN16580178	https://doi.org/10.6084/m9.figshare.16896877.v3
2^*d*^	3_1	UBA1723 sp002371265	15.2	96.37	3,549,890	320	15,986	41.1	98.28	0	SAMN16580181	GCA_018384995.1
MB2.44^*d*^	30_0	Novel species 30 within the genus SFDP01	11.6	NA	1,671,723	141	16,841	40.27	83.62	2.59	SAMN16580178	GCA_018385375.1
141^*d*^	31_0	Novel species 31 within the genus UBA7642	5.4	NA	816,795	374	2,327	43.69	74.14	3.43	SAMN16580182	GCA_018383255.1
24^*d*^	32_0	UBA636 sp002299675	7.0	98.61	1,489,909	499	3,818	38.2	87.77	0	SAMN16580181	GCA_018384855.1
MB2.95_2^*d*^	33_1	Novel species 33 within the genus UBA1067	16.3	NA	1,819,028	117	21,057	51.15	92.95	1.38	SAMN16580179	GCA_018384485.1
14	33_1	Novel species 33 within the genus UBA1067	NA	NA	1,974,080	586	4,278	51.27	87.3	5.52	SAMN16580180	https://doi.org/10.6084/m9.figshare.16896877.v3
81^*d*^	34_0	UMGS687 sp900544595	6.1	98.42	633,718	271	2,630	36.19	73.79	3.45	SAMN16580178	GCA_018384875.1
MB2.56^*d*^	35_0	SFEB01 sp004558105	18.2	98.26	987,321	154	7,610	45	89.83	1.72	SAMN16580178	GCA_018384135.1
157^*d*^	36_0	SFEL01 sp004557245	11.9	98.15	1,636,433	430	4,785	53.75	87.93	0.92	SAMN16580179	GCA_018383265.1
MBin.013^*d*^	37_0	Phil1 sp004558525	52.0	99.13	1,801,179	77	33,534	51.52	98.28	0	SAMN16580178	GCA_018384765.1
75^*d*^	38_0	Phil1 sp001940855	13.7	96.84	2,199,755	128	29,680	52.48	97.36	0.86	SAMN16580178	GCA_018384665.1
MB2.113^*d*^	39_1	Novel species 39 within the genus SFDB01	51.6	NA	1,791,970	22	134,508	48.31	98.28	0.86	SAMN16580178	GCA_018384315.1
MB2.89	39_1	Novel species 39 within the genus SFDB01	NA	NA	1,547,308	84	25,379	48.55	98.28	2.04	SAMN16580180	https://doi.org/10.6084/m9.figshare.16896877.v3
MB2.92^*d*^	4_1	*Fibrobacter* sp900142475	31.8	95.34	3,169,736	129	38,467	51.4	100	0	SAMN16580178	GCA_018384535.1
176	4_1	*Fibrobacter* sp900142475	NA	95.34	4,194,492	412	19,093	50.56	96.55	0.34	SAMN16580179	https://doi.org/10.6084/m9.figshare.16896877.v3
MB2.45	4_1	*Fibrobacter* sp900142475	NA	95.34	2,896,090	206	20,168	51.81	97.41	0	SAMN16580180	https://doi.org/10.6084/m9.figshare.16896877.v3
MB2.048	4_1	*Fibrobacter* sp900142475	NA	95.34	3,088,752	245	17,992	51.43	99.14	1.72	SAMN16580181	https://doi.org/10.6084/m9.figshare.16896877.v3
MB2.93	4_1	*Fibrobacter* sp900142475	NA	95.34	2,773,889	143	30,346	51.97	94.83	0	SAMN16580182	https://doi.org/10.6084/m9.figshare.16896877.v3
MB2.100^*d*^	40_0	SFDB01 sp004558825	14.5	99.08	1,551,443	95	27,719	51.3	98.28	0	SAMN16580179	GCA_018380645.1
MB2.73	41_1	Bifidobacterium pseudolongum	NA	99.54	1,668,768	92	25,976	63.66	98.28	0	SAMN16580178	https://doi.org/10.6084/m9.figshare.16896877.v3
MB2.115^*d*^	41_1	Bifidobacterium pseudolongum	19.7	99.54	1,641,528	98	28,425	63.73	98.28	0	SAMN16580179	https://doi.org/10.6084/m9.figshare.16896877.v3
33	41_1	Bifidobacterium pseudolongum	NA	99.54	1,927,110	344	8,852	63.35	100	2.07	SAMN16580180	https://doi.org/10.6084/m9.figshare.16896877.v3
MB2.005	41_1	Bifidobacterium pseudolongum	NA	99.54	1,653,031	115	22,636	63.74	94.83	0	SAMN16580181	https://doi.org/10.6084/m9.figshare.16896877.v3
MBin.057^*d*^	42_1	Novel species 42 within the genus *Oxalobacter*	15.4	NA	1,932,695	64	65,154	51.73	100	0	SAMN16580178	GCA_018384065.1
MB2.38_2	42_1	Novel species 42 within the genus *Oxalobacter*	NA	NA	1,758,046	155	15,110	51.88	94.83	0	SAMN16580182	https://doi.org/10.6084/m9.figshare.16896877.v3
71^*d*^	43_0	Novel species 43 within the genus *Oxalobacter*	5.1	NA	1,446,023	634	2,598	54.07	82.65	3.76	SAMN16580179	GCA_018383305.1
MB2.8^*d*^	44_1	Novel species 44 within the genus *Treponema*_D	27.0	NA	2,216,246	106	30,570	41.67	90.52	0	SAMN16580178	GCA_018384055.1
160	44_1	Novel species 44 within the genus *Treponema*_D	NA	NA	2,060,482	257	10,336	42.01	84.48	0	SAMN16580181	https://doi.org/10.6084/m9.figshare.16896877.v3
MB2.76^*d*^	45_0	Novel species 45 within the genus *Treponema*_D	76.3	NA	2,570,216	223	18,158	33.79	90.52	0.86	SAMN16580178	GCA_018385315.1
MB2.8_2^*d*^	46_0	Novel species 46 within the genus *Treponema*_D	126.9	NA	2,578,587	231	16,947	39.39	85.34	0	SAMN16580179	GCA_018383995.1
MB2.136	47_1	Novel species 47 within the genus *Treponema*_D	NA	NA	2,428,835	224	16,313	39.33	90.52	2.59	SAMN16580178	https://doi.org/10.6084/m9.figshare.16896877.v3
MB2.91_2^*d*^	47_1	Novel species 47 within the genus *Treponema*_D	25.0	NA	2,270,652	171	18,281	39.59	87.07	1.72	SAMN16580182	GCA_018385395.1
MB2.38^*d*^	48_0	Novel species 48 within the genus *Treponema*_D	51.9	NA	2,337,410	118	44,527	36.24	93.97	0	SAMN16580178	GCA_018385415.1
MB2.015^*d*^	49_0	Novel species 49_0 within the genus *Thiopseudomonas*	14.5	NA	2,246,815	124	28,600	57.9	87.15	0	SAMN16580181	GCA_018384595.1
107^*d*^	5_0	*Fibrobacter*_A sp002797675	6.1	99.08	2,165,544	690	3,858	49.86	89.66	2.57	SAMN16580182	GCA_018383235.1
226^*d*^	50_0	SFTJ01 sp004563195	NA	97.88	2,302,692	804	3,309	48.55	74.45	3.76	SAMN16580181	https://doi.org/10.6084/m9.figshare.16896877.v3
23^*d*^	51_0	CAG-279 sp004555955	15.6	98.07	2,104,689	723	3,468	49.91	86.83	0	SAMN16580180	GCA_018383405.1
102_2^*d*^	52_0	CAG-485 sp004553095	17.0	98.46	2,127,132	264	13,188	49.45	96.55	0	SAMN16580181	GCA_018384695.1
137^*d*^	53_1	*Sodaliphilus* sp004559845	7.9	97.27	1,991,868	565	4,337	49	86.05	3.76	SAMN16580179	GCA_018385115.1
59^*d*^	53_2	*Sodaliphilus* sp004559845	NA	98	2,158,008	537	5,560	48.6	82.76	3.45	SAMN16580181	https://doi.org/10.6084/m9.figshare.16896877.v3
60^*d*^	53_3	*Sodaliphilus* sp004559845	NA	97.66	1,970,221	708	3,237	48.91	74.69	0.16	SAMN16580182	https://doi.org/10.6084/m9.figshare.16896877.v3
109^*d*^	54_1	*Sodaliphilus* sp004557565	24.9	98.25	2,524,898	545	6,335	48.18	98.28	1.72	SAMN16580178	GCA_018385175.1
MB2.67	54_1	*Sodaliphilus* sp004557565	NA	98.25	1,797,432	321	6,002	48.53	72.41	0	SAMN16580179	https://doi.org/10.6084/m9.figshare.16896877.v3
166	54_1	*Sodaliphilus* sp004557565	NA	98.25	2,459,097	646	4,754	48.18	94.83	1.72	SAMN16580181	https://doi.org/10.6084/m9.figshare.16896877.v3
MB2.57_2	54_1	*Sodaliphilus* sp004557565	NA	98.25	1,813,601	326	5,991	48.11	84.48	1.72	SAMN16580182	https://doi.org/10.6084/m9.figshare.16896877.v3
157_2^*d*^	55_1	CAG-279 sp004561555	9.8	98.77	2,043,626	310	8,798	45.84	96.55	2.04	SAMN16580181	GCA_018384195.1
29	55_1	CAG-279 sp004561555	NA	98.77	2,007,650	358	7,577	46.49	89.66	6.21	SAMN16580182	https://doi.org/10.6084/m9.figshare.16896877.v3
MBin.037^*d*^	56_0	*Parabacteroides* sp000436495	23.4	96.8	3,220,218	162	30,201	42.39	90.52	0	SAMN16580178	GCA_018384275.1
102^*d*^	57_1	“*Prevotellamassilia*” sp004552865	7.3	97.48	2,025,313	673	3,832	53.09	88.4	4.47	SAMN16580182	GCA_018383295.1
97^*d*^	57_2	*Prevotellamassilia* sp004552865	NA	97.56	2,308,037	399	8,045	52.53	94.51	0	SAMN16580179	https://doi.org/10.6084/m9.figshare.16896877.v3
MB2.96^*d*^	58_0	UBA6398 sp003150315	8.1	98.63	1,741,886	284	7,383	45.21	78.84	3.28	SAMN16580182	GCA_018380595.1
MBin.002^*d*^	59_0	Novel species 59 within the genus *Caryophanon*	223.7	NA	2,149,313	170	25,329	42.61	90.75	1.88	SAMN16580180	GCA_018384745.1
61^*d*^	6_1	Novel species 6 within the genus UBA2658	16.5	NA	1,880,025	81	47,276	51.68	98.28	0	SAMN16580178	GCA_018385135.1
22	6_1	Novel species 6 within the genus UBA2658	NA	NA	1,744,341	272	8,568	51.82	92.87	0.86	SAMN16580179	https://doi.org/10.6084/m9.figshare.16896877.v3
MBin.007^*d*^	60_0	Bacteroides fragilis	98.0	99.03	5,507,632	223	142,876	43.24	100	6.9	SAMN16580178	https://doi.org/10.6084/m9.figshare.16896877.v3
155^*d*^	61_0	“*Bacteroides togonis*”	53.7	97.13	3,453,740	115	46,600	48.88	95.77	6.03	SAMN16580178	https://doi.org/10.6084/m9.figshare.16896877.v3
MB2.105^*d*^	62_0	Phocaeicola plebeius_A	33.1	96.01	3,369,676	79	59,934	44.69	93.03	0	SAMN16580178	https://doi.org/10.6084/m9.figshare.16896877.v3
MB2.88_2	63_1	*Prevotella* sp002251295	NA	99.34	2,564,796	199	20,555	46.6	90.05	0.69	SAMN16580178	https://doi.org/10.6084/m9.figshare.16896877.v3
MB2.76_3	63_1	*Prevotella* sp002251295	NA	99.34	2,415,843	137	30,218	46.8	96.55	2.76	SAMN16580179	https://doi.org/10.6084/m9.figshare.16896877.v3
MB2.128^*d*^	63_1	*Prevotella* sp002251295	32.1	99.34	2,299,847	120	30,440	46.82	92.07	1.72	SAMN16580181	GCA_018384355.1
MB2.81^*d*^	64_0	Prevotella hominis	48.5	95.41	2,612,864	325	10,261	43.46	79.31	5.5	SAMN16580182	GCA_018383955.1
MB2.133^*d*^	65_0	Jeotgalibaca porci	30.4	99.06	1,582,154	195	11,256	40.62	91.38	3.45	SAMN16580181	https://doi.org/10.6084/m9.figshare.16896877.v3
MBin.003	66_1	UBA4334 sp900316975	NA	97.68	2,468,196	282	15,587	48.26	96.55	3.61	SAMN16580178	https://doi.org/10.6084/m9.figshare.16896877.v3
MB2.31	66_1	UBA4334 sp900316975	NA	97.68	2,219,084	220	12,979	48.35	87.93	0.16	SAMN16580179	https://doi.org/10.6084/m9.figshare.16896877.v3
MB2.082^*d*^	66_1	UBA4334 sp900316975	30.8	97.68	2,254,860	180	19,020	48.38	91.03	0	SAMN16580181	GCA_018384255.1
MB2.6_2	66_1	UBA4334 sp900316975	NA	97.68	2,209,849	183	16,499	48.36	91.38	0	SAMN16580182	https://doi.org/10.6084/m9.figshare.16896877.v3
39^*d*^	67_0	Novel species 67 within the genus *Prevotellamassilia*	12.7	NA	2,200,295	355	9,893	53.4	96.55	3.45	SAMN16580178	GCA_018383375.1
100^*d*^	68_0	*Prevotella* sp002300055	28.1	99.35	2,972,104	166	30,510	53.78	98.28	3.45	SAMN16580179	GCA_018383335.1
122	69_1	UBA3388 sp004551865	NA	98.52	2,167,825	212	20,167	42.02	100	0	SAMN16580178	https://doi.org/10.6084/m9.figshare.16896877.v3
199_2	69_1	UBA3388 sp004551865	NA	98.52	2,285,050	332	11,249	42.35	98.28	2.04	SAMN16580179	https://doi.org/10.6084/m9.figshare.16896877.v3
MBin.021	69_1	UBA3388 sp004551865	NA	98.52	1,701,589	691	2,829	42.64	92.24	3.45	SAMN16580180	https://doi.org/10.6084/m9.figshare.16896877.v3
MB2.144^*d*^	69_1	UBA3388 sp004551865	102.4	98.52	1,912,382	96	30,760	42.28	100	0	SAMN16580181	GCA_018384335.1
MB2.48_2	69_1	UBA3388 sp004551865	NA	98.52	1,758,019	164	14,634	42.5	94.83	0	SAMN16580182	https://doi.org/10.6084/m9.figshare.16896877.v3
91^*d*^	7_1	Novel species 7 within the family UBA1242	17.9	NA	983,269	77	110,292	43.14	87.93	0	SAMN16580179	GCA_018384735.1
197	7_1	Novel species 7 within the family UBA1242	NA	NA	870,039	44	47,297	42.99	86.21	0	SAMN16580180	https://doi.org/10.6084/m9.figshare.16896877.v3
138^*d*^	70_1	UBA1232 sp004561775	10.2	97.13	1,592,739	433	5,120	47.32	93.1	0	SAMN16580178	GCA_018385195.1
142^*d*^	70_2	UBA1232 sp004561775	NA	96.78	1,360,217	578	2,641	47.6	71.47	0	SAMN16580180	GCA_018385055.1
138_2^*d*^	70_3	UBA1232 sp004561775	NA	97.4	1,411,325	552	2,921	47.81	78.45	1.88	SAMN16580182	https://doi.org/10.6084/m9.figshare.16896877.v3
36^*d*^	70_4	UBA1232 sp004561775	NA	96.92	1,601,980	467	4,579	47.58	94.67	4.31	SAMN16580181	https://doi.org/10.6084/m9.figshare.16896877.v3
MB2.79^*d*^	71_0	Novel species 71 within the genus RC9	10.2	NA	2,052,541	172	17,023	48.59	90.52	6.9	SAMN16580178	GCA_018384035.1
199^*d*^	72_1	*Prevotella* sp900548195	15.2	96.67	2,626,542	335	10,473	45.48	96.55	1.72	SAMN16580178	GCA_018384615.1
MB2.76_2	72_1	*Prevotella* sp900548195	NA	96.67	2,533,978	321	9,789	45.4	84.95	1.72	SAMN16580182	https://doi.org/10.6084/m9.figshare.16896877.v3
MB2.140	73_1	F082 sp900769945	NA	98.73	1,988,821	209	11,979	45.18	96.55	1.72	SAMN16580178	https://doi.org/10.6084/m9.figshare.16896877.v3
MB2.61	73_1	F082 sp900769945	NA	98.73	1,808,756	248	9,465	45.65	93.5	4.31	SAMN16580179	https://doi.org/10.6084/m9.figshare.16896877.v3
MB2.64_2	73_1	F082 sp900769945	NA	98.73	1,651,934	291	6,081	45.48	86	0	SAMN16580180	https://doi.org/10.6084/m9.figshare.16896877.v3
10_2^*d*^	73_1	F082 sp900769945	43.0	98.73	2,284,399	128	32,074	44.7	98.28	0	SAMN16580181	GCA_018384935.1
MB2.90^*d*^	74_0	Novel species 74 within the family *Bacteroidaceae*	18.2	NA	2,326,429	84	37,962	49.24	99.84	0	SAMN16580178	GCA_018385335.1
MB2.97^*d*^	75_1	RC9 sp004554455	17.3	98.49	1,454,040	207	8,934	55.36	81.9	5.17	SAMN16580178	GCA_018385325.1
MB2.52	75_1	RC9 sp004554455	NA	98.49	1,308,014	213	7,254	55.54	74.14	1.72	SAMN16580179	https://doi.org/10.6084/m9.figshare.16896877.v3
MB2.103	75_1	RC9 sp004554455	NA	98.49	1,276,739	206	6,872	55.35	72.41	1.8	SAMN16580181	https://doi.org/10.6084/m9.figshare.16896877.v3
MB2.109_2	75_1	RC9 sp004554455	NA	98.49	1,372,827	223	7,379	55.49	87.93	1.88	SAMN16580182	https://doi.org/10.6084/m9.figshare.16896877.v3
MB2.64^*d*^	76_0	RC9 sp000431015	12.6	98.71	2,153,407	220	13,730	53.78	85.34	2.04	SAMN16580178	GCA_018385295.1
MB2.118^*d*^	77_1	RC9 sp900546925	25.2	99	1,868,948	111	27,465	47.6	96.55	0	SAMN16580181	GCA_018384095.1
MB2.65	77_1	RC9 sp900546925	NA	99	2,033,531	160	20,061	47.63	98.28	6.9	SAMN16580182	https://doi.org/10.6084/m9.figshare.16896877.v3
136^*d*^	78_0	Novel species 78 within the genus UBA5920	9.5	NA	1,483,767	512	3,683	47.26	79.26	1.72	SAMN16580182	GCA_018385155.1
MB2.54_2^*d*^	79_1	RC9 sp004556005	70.3	98.85	1,821,683	170	17,759	51.99	92.24	6.03	SAMN16580179	GCA_018384575.1
MB2.087	79_1	RC9 sp004556005	NA	98.85	1,761,281	146	17,731	51.9	84.48	0.34	SAMN16580181	https://doi.org/10.6084/m9.figshare.16896877.v3
MB2.116	79_1	RC9 sp004556005	NA	98.85	1,674,035	172	15,264	51.82	80.44	1.72	SAMN16580182	https://doi.org/10.6084/m9.figshare.16896877.v3
MB2.54^*d*^	8_1	ER4 sp900317525	12.3	97.74	1,406,667	184	9,148	62.39	91.38	0	SAMN16580178	GCA_018384155.1
MB2.32_2	8_1	ER4 sp900317525	NA	97.74	1,436,706	192	8,977	62.3	88.32	1.72	SAMN16580179	https://doi.org/10.6084/m9.figshare.16896877.v3
MB2.101_2	8_1	ER4 sp900317525	NA	97.74	1,410,060	190	8,617	62.41	84.64	4.36	SAMN16580181	https://doi.org/10.6084/m9.figshare.16896877.v3
MB2.6^*d*^	80_1	RC9 sp000432655	92.5	97.68	1,524,799	45	52,040	49.03	94.83	0	SAMN16580178	GCA_018385255.1
MB2.35	80_1	RC9 sp000432655	NA	97.68	1,591,216	87	35,010	49.13	86.83	0	SAMN16580179	https://doi.org/10.6084/m9.figshare.16896877.v3
MB2.052	80_1	RC9 sp000432655	NA	97.68	1,493,030	57	48,600	49.11	86.21	4.31	SAMN16580181	https://doi.org/10.6084/m9.figshare.16896877.v3
MB2.5	81_1	RC9 sp004552965	NA	98.74	1,923,243	176	15,583	53.71	91.38	0	SAMN16580178	https://doi.org/10.6084/m9.figshare.16896877.v3
MB2.88^*d*^	81_1	RC9 sp004552965	42.0	98.74	1,853,595	164	17,205	53.7	88.79	1.72	SAMN16580179	GCA_018384515.1
MB2.058	81_1	RC9 sp004552965	NA	98.74	1,938,889	163	15,807	53.7	93.97	3.45	SAMN16580181	https://doi.org/10.6084/m9.figshare.16896877.v3
MB2.101	81_1	RC9 sp004552965	NA	98.74	1,930,449	172	15,422	53.8	93.1	1.72	SAMN16580182	https://doi.org/10.6084/m9.figshare.16896877.v3
MB2.9^*d*^	82_1	Novel species 82 within the genus RC9	41.5	NA	1,687,862	116	23,135	54.82	93.1	7.37	SAMN16580179	GCA_018384555.1
MB2.83_3	82_1	Novel species 82 within the genus RC9	NA	NA	1,802,719	123	19,776	54.82	86.21	1.38	SAMN16580182	https://doi.org/10.6084/m9.figshare.16896877.v3
MB2.26	83_1	Novel species 83 within the genus RC9	NA	NA	1,582,938	169	13,745	54.89	73.67	4.39	SAMN16580179	https://doi.org/10.6084/m9.figshare.16896877.v3
MB2.52_2	83_1	Novel species 83 within the genus RC9	NA	NA	1,427,062	226	7,651	55.05	70.97	1.25	SAMN16580180	https://doi.org/10.6084/m9.figshare.16896877.v3
MB2.109^*d*^	83_1	Novel species 83 within the genus RC9	18.1	NA	1,694,760	169	15,584	54.83	87.93	0.86	SAMN16580181	GCA_018384235.1
MB2.77	83_1	Novel species 83 within the genus RC9	NA	NA	1,660,044	174	13,126	54.89	87.07	0.16	SAMN16580182	https://doi.org/10.6084/m9.figshare.16896877.v3
MB2.48^*d*^	84_0	Novel species 84 within the genus RC9	6.8	NA	1,027,778	198	5,726	53.89	70.38	3.45	SAMN16580178	GCA_018384215.1
MB2.029^*d*^	85_1	Novel species 85 within the genus *Myroides*	53.3	NA	1,924,990	331	6,245	34.49	76.15	2.04	SAMN16580181	GCA_018384465.1
MB2.8_3	85_1	Novel species 85 within the genus *Myroides*	NA	NA	1,391,904	314	4,675	34.89	73.82	3.92	SAMN16580182	https://doi.org/10.6084/m9.figshare.16896877.v3
MB2.020^*d*^	86_0	Novel species 86_0 within the genus YIM-102668	49.0	NA	2,770,569	206	20,377	31.23	93.1	2.59	SAMN16580181	GCA_018384435.1
170	87_1	Streptococcus alactolyticus	NA	99.2	1,793,166	138	24,966	40.54	98.28	2.59	SAMN16580178	https://doi.org/10.6084/m9.figshare.16896877.v3
MB2.24^*d*^	87_1	Streptococcus alactolyticus	259.0	99.2	1,460,880	63	42,702	41.11	93.97	0	SAMN16580179	https://doi.org/10.6084/m9.figshare.16896877.v3
MB2.83_2	87_1	Streptococcus alactolyticus	NA	99.2	1,591,166	125	16,988	41.03	97.81	4.47	SAMN16580180	https://doi.org/10.6084/m9.figshare.16896877.v3
MB2.097	87_1	Streptococcus alactolyticus	NA	99.2	1,460,509	80	30,127	40.95	87.07	3.45	SAMN16580181	https://doi.org/10.6084/m9.figshare.16896877.v3
MB2.13	87_1	Streptococcus alactolyticus	NA	99.2	1,514,600	85	25,521	40.92	96.55	0	SAMN16580182	https://doi.org/10.6084/m9.figshare.16896877.v3
MB2.32^*d*^	88_1	Limosilactobacillus reuteri	68.5	96.32	1,596,378	71	39,896	38.8	94.83	0	SAMN16580178	https://doi.org/10.6084/m9.figshare.16896877.v3
MB2.34	88_1	*Limosilactobacillus reuteri*	NA	96.32	1,650,438	151	15,523	38.84	95.61	0.57	SAMN16580180	https://doi.org/10.6084/m9.figshare.16896877.v3
MB2.39	88_1	*Limosilactobacillus reuteri*	NA	96.32	1,480,767	110	19,594	39.07	91.69	0	SAMN16580182	https://doi.org/10.6084/m9.figshare.16896877.v3
MBin.004	89_1	Lactobacillus amylovorus	NA	97.64	1,945,777	267	15,066	37.88	89.66	1.72	SAMN16580178	https://doi.org/10.6084/m9.figshare.16896877.v3
MB2.138^*d*^	89_1	Lactobacillus amylovorus	26.1	97.64	1,515,149	119	19,534	38.63	98.28	0	SAMN16580181	https://doi.org/10.6084/m9.figshare.16896877.v3
MB2.95^*d*^	9_1	Novel species 9 within the genus ER4	21.8	NA	1,237,038	125	12,747	58.23	93.89	0	SAMN16580178	GCA_018380655.1
MB2.84	9_1	Novel species 9 within the genus ER4	NA	NA	1,265,700	132	12,202	58.34	94.83	3.03	SAMN16580179	https://doi.org/10.6084/m9.figshare.16896877.v3
MB2.105_2	9_1	Novel species 9 within the genus ER4	NA	NA	1,203,093	162	8,475	58.28	88.35	1.02	SAMN16580182	https://doi.org/10.6084/m9.figshare.16896877.v3
MB2.91^*d*^	90_0	*Methanobrevibacter*_A sp900769095	39.3	98.84	2,058,317	258	10,227	32.03	92.52	6.07	SAMN16580178	GCA_018385235.1
9^*d*^	91_0	CAG-177 sp900771185	5.8	95.19	1,339,283	491	3,126	49.69	78.84	3.45	SAMN16580179	GCA_018385015.1
MB2.102^*d*^	92_0	Novel species 92 within the genus UMGS1384	12.7	NA	2,110,823	63	51,290	54.68	87.93	0	SAMN16580178	GCA_018384365.1
MBin.083	93_1	Acinetobacter pseudolwoffii	NA	98.51	3,218,104	880	5,371	44.46	92.71	4.31	SAMN16580178	https://doi.org/10.6084/m9.figshare.16896877.v3
MB2.58^*d*^	93_1	Acinetobacter *pseudolwoffii*	25.6	98.51	2,150,411	294	8,889	44.18	86.13	0	SAMN16580179	https://doi.org/10.6084/m9.figshare.16896877.v3
MB2.66^*d*^	94_1	Escherichia flexneri	35.1	97.97	3,512,380	515	8,317	50.96	86.21	0	SAMN16580178	https://doi.org/10.6084/m9.figshare.16896877.v3
169	94_1	Escherichia *flexneri*	NA	97.97	3,947,168	844	6,167	51.15	94.67	3.45	SAMN16580180	https://doi.org/10.6084/m9.figshare.16896877.v3
15	94_1	Escherichia *flexneri*	NA	97.97	3,212,344	1,334	2,664	51.49	76.77	1.72	SAMN16580181	https://doi.org/10.6084/m9.figshare.16896877.v3
106	94_1	Escherichia *flexneri*	NA	97.97	2,360,607	1,127	2,237	51.91	79.7	2.85	SAMN16580182	https://doi.org/10.6084/m9.figshare.16896877.v3
159^*d*^	95_0	Novel species 95 within the genus SFVR01	19.9	NA	1,795,205	360	7,175	37.19	98.28	4.31	SAMN16580178	GCA_018385185.1
4^*d*^	96_0	Psychrobacter pasteurii	9.7	97.62	2,133,879	600	4,479	43.11	80.25	3.45	SAMN16580181	https://doi.org/10.6084/m9.figshare.16896877.v3
144^*d*^	97_1	Novel species 97 within the genus Rs-D84	5.7	NA	734,502	267	3,229	44.87	81.58	0	SAMN16580178	GCA_018383165.1
82^*d*^	97_2	Novel species 97 within the genus Rs-D84	NA	NA	769,008	165	5,928	44.97	86.05	1.72	SAMN16580182	https://doi.org/10.6084/m9.figshare.16896877.v3
146^*d*^	97_3	Novel species 97 within the genus Rs-D84	NA	NA	855,668	89	15,967	44.74	91.38	1.72	SAMN16580179	https://doi.org/10.6084/m9.figshare.16896877.v3
10^*d*^	98_0	MX-02 sp006954405	9.2	99.14	1,434,374	143	17,966	47.98	97.66	1.4	SAMN16580178	GCA_018384975.1
150^*d*^	99_0	UBA4372 sp900766785	14.0	97.69	2,081,807	432	6,161	50.82	77.66	0	SAMN16580182	GCA_018385095.1

a Genome statistics were derived using CheckM. NA, not applicable.

b Species and strain clusters have been numbered according to clustering at 95% and 99% ANI, with the species cluster number followed by an underscore and then the strain designation.

c The taxonomic assignment represents the species designation given by the GTDB toolkit. For novel species, we have listed our species cluster within the lowest level assigned by GTDB.

d Strain representative with the best binning statistics.

### Data availability.

The shotgun sequence data have been deposited at the NCBI under BioProject accession number PRJNA672868 and BioSample accession numbers SAMN16580178, SAMN16580179, SAMN16580180, SAMN16580181 and SAMN16580182, with supplementary information on Figshare at https://doi.org/10.6084/m9.figshare.16896877.v3. The recovered MAGs dereplicated at 99% ANI can be found on Figshare at https://doi.org/10.6084/m9.figshare.16896877.v3, with the assemblies representing novel species available under NCBI BioProject accession number PRJNA672868 (Table 1).
